# High-Oleic Sunflower Oil as a Potential Substitute for Palm Oil in Sugar Coatings—A Comparative Quality Determination Using Multispectral Imaging and an Electronic Nose

**DOI:** 10.3390/foods13111693

**Published:** 2024-05-28

**Authors:** Nicole Ollinger, Bernhard Blank-Landeshammer, Lisa Schütz-Kapl, Angeline Rochard, Iris Pfeifenberger, Jens Michael Carstensen, Manfred Müller, Julian Weghuber

**Affiliations:** 1 FFoQSI–Austrian Competence Centre for Feed and Food Quality Safety & Innovation FFoQSI GmbH, Technopark 1D, 3430 Tulln, Austria; bernhard.blank-landeshammer@fh-wels.at (B.B.-L.);; 2 Center of Excellence Food Technology and Nutrition, University of Applied Sciences Upper Austria, Stelzhamerstrasse 23, 4600 Wels, Austria; 3 Videometer A/S, Horkaer 12B, 3rd floor, DK-2730 Herlev, Denmark; jmc@videometer.com; 4 Puratos Austria GmbH, Maria-Theresia-Straße 41, 4600 Wels, Austria; mmueller@puratos.com

**Keywords:** multispectral imaging, electronic nose, acid value, food quality, palm oil, sunflower oil

## Abstract

Palm oil has a bad reputation due to the exploitation of farmers and the destruction of endangered animal habitats. Therefore, many consumers wish to avoid the use of palm oil. Decorative sugar contains a small amount of palm oil to prevent the sugar from melting on hot bakery products. High-oleic sunflower oil used as a substitute for palm oil was analyzed in this study via multispectral imaging and an electronic nose, two methods suitable for potential large-batch analysis of sugar/oil coatings. Multispectral imaging is a nondestructive method for comparing the wavelength reflections of the surface of a sample. Reference samples enabled the estimation of the quality of unknown samples, which were confirmed via acid value measurements. Additionally, for quality determination, volatile compounds from decorative sugars were measured with an electronic nose. Both applications provide comparable data that provide information about the quality of decorative sugars.

## 1. Introduction

Palm oil is a very versatile raw product in the food industry. Although the ratio of unsaturated-to-saturated fatty acids is regarded as beneficial, as well as the fact that the oil contains many antioxidants, this approach has been heavily criticized due to the cultivation conditions and the deprivation of the habitat of endangered species [1].

Oil palms are grown in more than 40 countries, almost all of which are located in Southeast Asia [2,3,4].

Palm oil production has a negative impact on biodiversity. In particular, large plantations rely on monocultures for higher yields. Furthermore, palm oil production increases carbon emissions due to the reduction in peatland and wet land, which leads to habitat loss [5].

Oil palm is the highest-yielding vegetable oil crop, with 3.3 metric tons of palm oil per hectare reported in the year 2020, followed by coconut, sunflower and rapeseed, each with 0.7 tons per hectare [6].

Although the demand for plant oils is rising, the crop delivering the highest yield is rejected by consumers. The reason for such decisions is often due to certifications and labels such as ‘palm oil-free’, which mislead the consumer. Furthermore, palm oil is often used in highly processed food, which suggests that it is necessary to avoid the intake of palm oil to remain healthy, even though there is a lack of scientific evidence regarding this conclusion [2,5].

Because palm oil is avoided by customers, the industry is seeking alternatives to this versatile food. Therefore, various alternative vegetable oils have been evaluated for their suitability in terms of sensory and organoleptic properties, as well as storage stability. The time- and cost-saving execution of storage trials and shelf-life tests require high-throughput analysis methods.

Decorative sugars on pasties contain, among other additives, vegetable oils such as palm oil, which prevents the melting of sugar from hot baked goods. In this study, we compared devices for shelf-life tests of vegetable oils containing decorative sugars because traditional acid value determination is time-consuming and not suitable when a larger dataset of information is required. For this purpose, the quality of the selected amounts of differently stored and spoiled samples was examined using acid value determination and subsequently measured with the respective devices.

Two devices were tested with high-oleic sunflower oil sugar and palm oil sugar to determine their suitability for quality prediction. The first device was a multispectral imaging analyzer that measures the samples in a nondestructive manner by illuminating the sample via 19 wavelengths and comparing the obtained data with reference samples. By using multivariate analysis algorithms in conjunction with this device, a percentage quality assessment can be made in relation to the reference values. The second device was an electronic nose based on dual flash gas chromatography (GC) with a headspace autosampler that allows for sensitive detection of volatile components. This method also requires the measurement of reference samples in advance, which are subsequently used to estimate the quality. The advantage of the electronic nose over conventional GC systems is that the software offers a fast yes-or-no discrimination program that is suitable for use with untrained persons. Trained individuals must perform initial calibrations of the software beforehand for both devices. For comparison purposes, selected samples were also measured using gas chromatography-mass spectrometry.

The hypothesis of this study was that high-oleic-acid sunflower seed oil is an appropriate alternative to palm oil due to its subtle taste and favorable chemical composition.

## 2. Materials and Methods

### 2.1. Decorative Sugar Preparation

To ensure the absence of substances that may have an uncontrolled influence on the stability of the products, all of the test samples were freshly prepared by mixing saccharose with 1.5% of the respective vegetable oil. All of the samples (sugars and oils) as well as the references that were beyond the “best before” date were obtained from Puratos GmbH (Wels, Austria). To ensure that the used oils can be considered representative, fatty acid methyl ester analyses of fresh high-oleic sunflower and palm oil were determined and compared to reference values from CODEX STAN 210-1999, as shown in Table 1.

### 2.2. Oil Extraction from Decorative Sugars and Evaporation of Solvents

All of the chemicals were purchased from Bartelt (Graz, Austria) unless otherwise stated. The decorative sugars containing 1.5% oil were weighed into 33 × 100 mm cellulose extraction sleeves from VWR (Darmstadt Germany) and hot-extracted with 150 mL of petrol ether (40–60) for 1 h. The solvent was completely removed via evaporation in an IKA RV 8 rotary evaporator at 65 °C (Staufen, Germany).

### 2.3. Acid Value Determination

Acid value determination was performed according to the standard method of phenolphthalein titration [7]. The oil was weighed and mixed with a 1 + 1 mixture of toluene + ethanol to a final concentration of 0.03% phenolphthalein as an indicator. An additional 100 µL of 3% phenolphthalein was added, and the mixture was stirred. The acid content was determined via titration with 0.01 M potassium hydroxide volumetric standard solution and calculated.

### 2.4. Multispectral Imaging Sample Preparation

The sugar samples for multispectral imaging were stored at room temperature for approximately half a year to induce rancidity and determine the stability of the high-oleic sunflower seed oil. Aliquots were taken at irregular time intervals. Palm oil sugar was previously tested and excluded from this long-term test due to the very long stability of the product.

### 2.5. Multispectral Imaging

The VideometerLab from Videometer A/S (Herlev, Denmark) is a multispectral image capture tool combining 19 different wavelengths between 365 nm and 970 nm. This tool consists of a matte white sphere with numerous light-emitting diodes (LEDs) for each wavelength, as well as a monochrome camera situated on top of the device for taking pictures. To ensure that all the LEDs could achieve the optimal dynamic range, the device requires a radiometric and geometric calibration, as well as a light setup, before pictures can be acquired [8].

**Figure 1:**
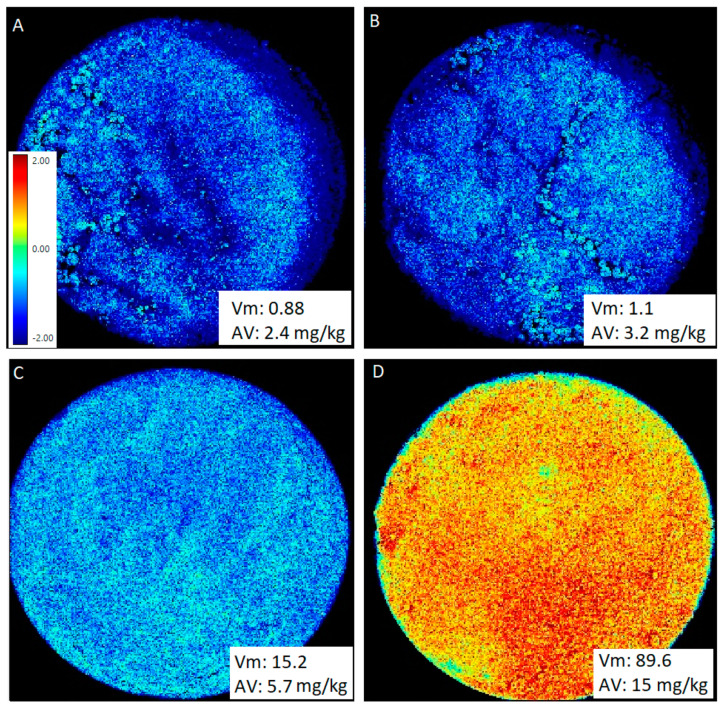
Calibration of the VideometerLab multispectrometer via normalized canonical discriminant analysis of sugar coatings with high-oleic sunflower seed oil. (**A**) shows that the fresh sugar coating is marked blue and is thereby classified as having a negative value. The rancidity index of the Videometer measurement was 0.88 and the acid value was 2.4 mg/kg. (**B**) is slightly more rancid, with a Videometer value of 1.1 and an acid value of 3.2 mg/kg. (**C**) shows the nCDA of a sugar/oil sample with a Videometer value of 15.2 and an acid value of 5.7 mg/kg. (**D**) represents s rancid sample, which is marked red and orange and is thereby classified as having a positive value with a Videometer value of 89.6 and an acid value of 15 mg/kg.

For measurements, the samples were poured into 9 cm petri dishes and flattened to prevent shadows. Afterward, the plates were placed under a sphere, which was lowered onto the sample. Multispectral images were captured with a resolution of approximately 30 µm per pixel. Each pixel that was captured by the camera represents a surface reflection, thus enabling measurement of the texture, color, and chemical differences of the sample surface. The entire reflectance spectrum in every pixel can be mapped onto a chemical abundance value for that pixel, and the abundance values for all pixels represent an abundance map as illustrated in Figure 1. The conversion of a 19-dimensional reflectance spectrum to a univariate abundance map is referred to as a transformation. This transformation is learned from a set of training images, and the type of transformation is termed normalized canonical discriminant analysis (nCDA). It combines the well-established canonical discriminant analysis (CDA), which estimates the linear mapping that optimizes the Rayleigh quotient among class variance over the within-class variance with an optimization of hyperparameters that chooses optimal normalization and basis expansion before performing a regular CDA. The nCDA map can then be used as an abundance map for a trained chemical difference between fresh and rancid samples.

### 2.6. Electronic-Nose Sample Preparation

Four grams of the sugars modified with palm oil or high-oleic sunflower oil and pure oil were weighed in a 20 mL headspace vial from Winopal (Elze, Germany) and sealed under a hood. The oils and sugar mixtures were subjected to accelerated shelf-life testing in a dry chamber at 50 °C for two weeks. All of the samples were kept in the dark during storage.

High oleic-acid concentrations in sunflower oil and palm oil were measured to compare the initial volatile compounds. Mixtures of saccharose and oils were compared, and aroma profiles were determined. For a time period of two weeks, the samples were stressed at 50 °C to induce rancid flavors. Once a week, the measurements were conducted in triplicate.

### 2.7. Electronic-Nose Measurements

For the determination of aroma compounds using the Heracles Neo e-nose from Alpha Mos (Toulouse, France), all of the samples were incubated at 80 °C for 20 min. Subsequently, headspace injection with a 5.000 µL volume at 70 °C syringe temperature with 125/µLs injection speed was performed, and trapping temperature was set to 40 °C for 50 s. Gas injection was automatically performed. The oven temperature was set to 50 °C, and the heating rate was increased from 3 °C/s to 250 °C/s. The columns MXT 5 (Crossbond 5% diphenyl/95% dimethyl polysiloxane; 10 m, 0.18 mm internal diameter, 0.4 µm film thickness) and MXT 1701 (Crossbond 14% cyanopropylphenyl/86% dimethyl polysiloxane; 10 m, 0.18 mm internal diameter, 0.4 µm film thickness) were used in parallel for better discrimination and resolution of the test substances. Two flame ionization detectors (FIDs) were used for recording.

Instrument operation and data analysis were performed by using Alphasoft software release 2021 and the AroChemBase database version 8 from Alpha Mos (Toulouse, France). PCA was performed by using the Alphasoft software; moreover, for better visibility, automatic annotation was increased by using CorelDraw GraphicsSuite 2019. The software automatically calculates the discrimination index, which allows for the measurement of the separation of groups. It indicates that two groups are statistically significant with a value above 90. The discrimination index can be negative when two groups are overlaid.

For the evaluation of the data, small peaks below 1000 counts per area were deleted to reduce background noise. The peak areas were determined to obtain relative quantities.

To compare the measurement results of both columns, the Kovats retention index was used to transform the retention times into column-independent constants. Kovats calibration was performed with a mixture of n-alkanes from C6-C16 to compare the relative retention times of both columns with those of the substances in the database for identification. Based on the retention time index, the most plausible substances were taken from AgroChemBase by excluding all datasets with a relevance index below 97 after applying a filter for the application field ‘Food’. As the identified substances varied for the triplicates of the same sample, no further attempts at substance identification were made.

### 2.8. Headspace GC-MS Analysis of the Sugar Coatings

GC-MS headspace analysis was performed using a Trace 1300 gas chromatograph coupled to an ISQ QD single-quadrupole mass spectrometer equipped with a PTV injector and a TriPlusRSH headspace autosampler from Thermo Scientific (Waltham, MA, USA). Four grams of each sample was transferred to 15 mL headspace vials and incubated at 80 °C for 20 min under agitation, and 2.0 mL of the headspace was aspirated via an autosampler syringe, which was heated to 70 °C. The injector temperature was kept constant at 240 °C, and the samples were injected in splitless mode onto a Stabilwax-DA column (30 m × 0.25 mm, 0.25 μm film thickness) from Restek (Centre County, PA, USA). Helium was used as the carrier gas at a constant flow rate of 1.0 mL/min. The oven temperature was kept constant at 35 °C for 3 min, after which it was increased to 140 °C at 8 °C/min and further increased to 220 °C at 25 °C/min, followed by a constant period of 2 min at 220 °C before returning to the initial conditions. The injector and ion-source temperatures were set to 240 °C and 200 °C, respectively. Full scans from *m*/*z* 30–350 were recorded at a rate of 8 scans/s. Instrument operation and data analysis were performed by using the Chromeleon 7.2 software package from Thermo Scientific (Waltham, MA, USA), and analytes were tentatively identified using the NIST 11 spectral library in conjunction with the NIST 11 GC RI database.

### 2.9. Fatty Acid Methyl Ester Analysis of Pure Oils by GC-MS

GC-MS analysis was used to ensure that the oils were of good quality and that they contained the expected fatty acid composition. Fatty acids were converted to their respective methyl esters (FAMEs) following a two-step trans-esterification protocol according to ISO 12966-2:2017 [9], with minor modifications. The extracted lipids were mixed with 200 µL of 0.2 M sodium methoxide from Alfa Aesar (Thermo Fisher Scientific, Heysham, UK) and incubated at 60 °C for 45 min with shaking. Subsequently, 70 µL of 1 M methanolic sulfuric acid was added, and the mixture was incubated at 60 °C for an additional 30 min. The FAME was extracted by the addition of 500 µL of n-hexane and 600 µL of sodium chloride, and an aliquot of the hexane phase was subjected to GC-MS analysis, as described previously [10]. A trace 1300 gas chromatograph coupled to an ISQ 7000 single-quadrupole mass spectrometer with a PTV injector and equipped with a TR-FAME column (60 m × 0.25 mm, 0.25 μm film thickness, all Thermo Scientific, Waltham, MA, USA) was used. The injector temperature was kept constant at 240 °C with a split ratio of 1/50 and a flow rate of 1.5 mL/min of helium. The initial oven temperature was held at 55 °C for 2 min, after which it was increased to 190 °C at a rate of 20 °C/min and further increased to 240 °C at 3 °C/min, which was maintained for 4 min. The injector and ion-source temperatures were set to 240 °C and 250 °C, respectively. Full scans from *m*/*z* 40–400 were recorded, and SIM scans at *m*/*z* 55, 67, 74 and 79 were used for quantification with the Supelco 37 Component FAME Mix from Sigma-Aldrich (St. Louis, MO, USA) as an external standard.

### 2.10. Statistical Analyses

Acid values were calculated in Excel, and the data were analyzed in GraphPad Prism 8.

The VideometerLab software version 3.22 (Herlev, Denmark) was used to perform the multispectral imaging data analyses. Fresh samples and rancid sugars were marked separately as regions of interest (ROIs). The process of transforming multispectral images (MSIs) involved performing normalized canonical discriminant analysis (nCDA) to determine the effect of rancidity on decorative sugars. A simple threshold was used, with positive values (red) representing rancid and negative values (blue) representing not rancid. Finally, the degree of rancidity was determined for the various sample plates in an Excel file. Correlations were evaluated in GraphPad Prism 8, and the data were obtained from the electronic nose.

Pictures were adjusted and labeled in CorelDraw GraphicsSuite 2019.

## 3. Results

### 3.1. Positive Correlation between Multispectral Imaging and the Acid Concentration Determined by Titration

The suitability of multispectral imaging for rancidity determination was tested. Aged- and fresh-sugar samples mixed with high-oleic sunflower seed oil were obtained by the company partner. Data from palm oil samples were excluded from this study, as previous data have proven the long-term stability of the oil. The palm oil samples were always blue (fresh) and labeled via the software, even after long storage periods; therefore, they were not suitable for comparative measurements, as palm oil is stable for a long period of time. The high-oleic sunflower oil samples were stored for half a year at room temperature. At irregular intervals, samples were taken and measured via acid value determination and multispectral imaging analysis on the same day.

**Figure 2:**
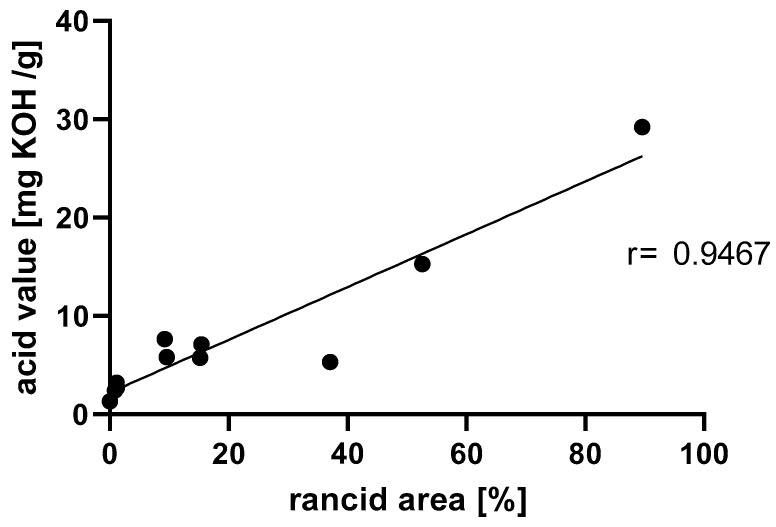
Proof of the correlation between the acid content determined by conventional Soxhlet extraction with that determined by subsequent titration and that determined by multispectral imaging analysis via a VideometerLab. The value that was obtained from the multispectral imaging is shown on the x-axis, and the result of the acid value determination of the same sample at the same time is shown on the y-axis. Pearson r = 0.9467.

Pictures from a spoiled mixture of high-oleic sunflower oil and sugar (acid: 15 mg/kg) and a fresh sample (acid: 0.26 mg/kg) were taken as two references for nCDA analysis (Figure 1). The combination of the two pictures was used in a session to assess the quality of 10 other samples. Figure 1 shows four representative pictures of the nCDA analysis and different spoilage states. All of the sugar/oil mixtures were Soxhlet-extracted to obtain pure oil for subsequent acid value determination. The correlation analysis showed a linear correlation with all of the other samples, as shown in Figure 2. Calibration of the device with fresh decorative sugars and with sugars that exceeded the limit of rancidity demonstrated that the correlation provided a reliable calibration line. Higher amounts of spoiled sugars, which are not suitable for consumption, had very high values for both measurements and were located out of the linear range.

For the correlation of lipid extraction for acid value determination via titration and multispectral imaging, twelve aged saccharose and high-oleic sunflower oil samples, including positive and negative reference samples, were analyzed at irregular time intervals. The acid value was determined by titration and, as expected, resulted in low values for fresh samples and high values for rancid samples. The VideometerLab measurements for the samples were carried out on the same day as the acid value determination. The device determined the rancidity of the sample as a percentage of the surface area. As shown in Figure 1A, we obtained an acid value of 2.4 mg/kg, and a rancidity value of 0.88% from the VideometerLab analysis for a fresh sample. Furthermore, a highly rancid sample with an acid value of 15 mg/kg resulted in a rancidity of 89.6% in the VideometerLab analysis (Figure 1D). The high value of 2.4 mg/kg even in a fresh sample is not surprising, because the oil to be measured was obtained by Soxhlet extraction and was therefore already intensively exposed to oxygen and heat. The suitability of the device for determining the rancidity of a foodstuff has already been demonstrated in a study with butter cookies [11] and with sunflower seeds [12].

The extracted and measured oil content in the mixtures was 0.83% ± 0.57, which was slightly below the initial amount of 1.5%.

### 3.2. The Volatile Profile Differed between Fresh and 2-Week-Old Fresh Sunflower Oil and Palm Oil

**Figure 3:**
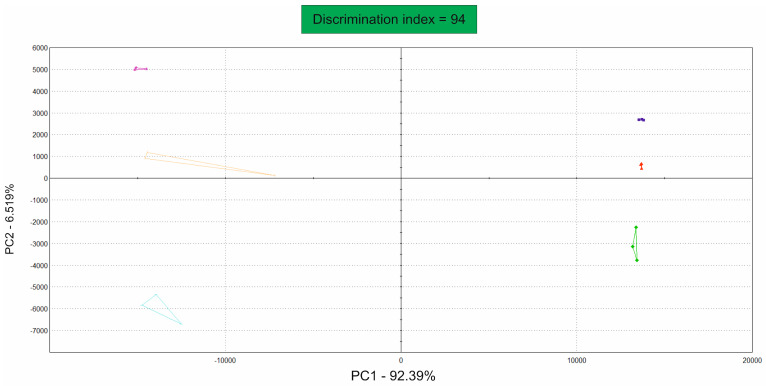
PCA of high-oleic-acid content for sunflower oil and palm oil. The three bright triangles on the left-hand side of the y-axis represent the triplicates of high-oleic sunflower oil, with the freshest sample at the top (purple) and the oldest sample at the bottom (cyan). On the right-hand side, the three darker triangles of the palm oil are shown with the fresh sample at the top (dark blue) and the oldest sample at the bottom (dark green). The discrimination index was 94, which means that the groups are statistically different, with PC1 accounting for 92.39% and PC2 accounting for 6.519%.

High-oleic sunflower oil and palm oil were measured fresh and after one and two weeks of heat stress at 50 °C in a sealed headspace vial, in triplicate. Based on the aroma profile, the oil types were able to be distinguished from each other; additionally, the volatile substances differed depending on the storage time, as indicated by the PCA in Figure 3.

The high-oleic sunflower oil samples are presented on the negative quadrants of the x-axis, and the palm oil sample is presented on the positive quadrants. For both oils, the fresh samples were in the higher range of the y-axis, the one-week stored oils were close to the x-axis, and the two-week heated samples were in the negative range of the y-axis. All of the samples were measured in triplicate. Outliers such as the high-oleic sunflower oil samples after one week of incubation (light green) can occur due to minor naturally occurring irregularities or weight differences. Nevertheless, the high discrimination index of 94 indicated that no group was superposed with another.

### 3.3. The Volatile Components of Fresh Sugar Mixtures Are Equal and Become Different over Time

**Figure 4:**
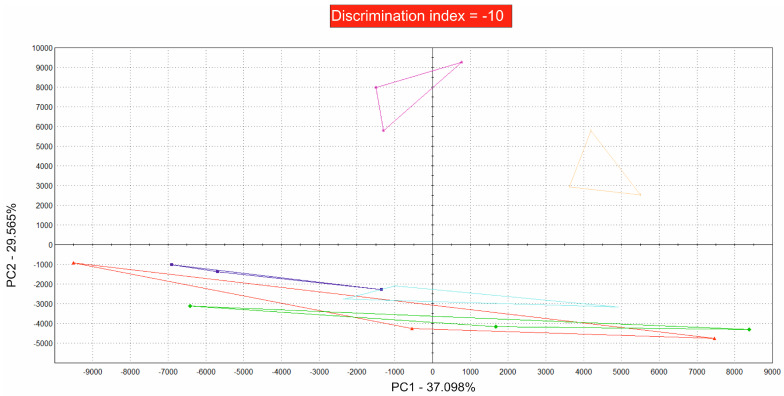
PCA of volatile compounds of saccharose mixed with high-oleic sunflower oil and palm oil. High-oleic sunflower oil/sugar fresh: purple; high-oleic sunflower oil/sugar one week at 50 °C: bright orange; high-oleic sunflower oil/sugar two weeks at 50 °C: turquoise; palm oil/sugar fresh: dark blue; palm oil/sugar at 50 °C for one week: red; palm oil/sugar at 50 °C for two weeks: green. The discrimination index was −10, which means that the groups are at least partly overlaying, with 37.098% for PC1 and 29.565% for PC2.

Fresh and briefly stored sugar/oil mixtures are hardly distinguishable from each other. Therefore, the discrimination index was negative, which indicates that the sample groups were at least partly overlapping and therefore indistinguishable within the first two weeks, although the oils alone were different (Figure 4). Figure 4 shows the results for the mixtures of fresh palm oil and high-oleic sunflower oil after one week and after two weeks of storage at 50 °C.

**Figure 5:**
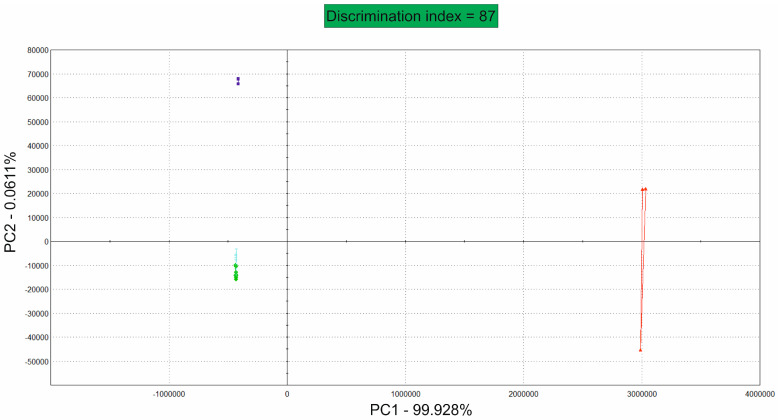
PCA of volatile compounds. Saccharose sunflower-seed oil mixtures (turquoise) and saccharose palm oil (light green) mixtures are located in the lower left corner. Long-term stressed high-oleic sunflower samples were collected at large distances. In blue, a sample was stored for 2.5 months (0.29 ± 0.03 mg KOH/mg oil) at −18 °C, and the same sample was stored at room temperature for 2.5 months (4.05 ± 0.06 mg KOH/mg oil), in red. The discrimination index was 87, which means that the data are not overlaying (without significant difference), with a PC1 of 99.928% and a PC2 of 0.06117%.

The samples became more distinguishable as the degree of spoilage increased. Figure 5 presents the same saccharose high-oleic sunflower oil mixtures and the saccharose palm oil mixtures. Additionally, 2.5-month-old saccharose/high-oleic sunflower oil samples stored at room temperature were added. The indistinguishable data for the fresh samples are presented in the lower-left quadrant, and the data for the samples stored at −18 °C for 2.5 months are shown in the upper-left quadrant. The 2.5-month-old saccharose/high-oleic sunflower oil samples shown in red are the same samples that were measured via multispectral imaging analyses; these samples served as the ‘spoiled reference’.

The blue sample was stored for 2.5 months at −18 °C and had an acid value of 0.29 mg KOH/mg oil, which did not exceed the acceptable value of 0.6. The sample stored at room temperature for the same time period had an acid value of 4.05 ± 0.06 mg KOH/mg oil and was considered to be rancid. The results of the VideometerLab PCA confirmed these findings.

### 3.4. Analysis of Methyl Esters and Acid Value Determination in High-Oleic Sunflower Oil and Palm Oil

The aroma profiles of pure fresh high-oleic-acid sunflower oil and fresh palm oil were compared to determine the initial differences between both products. To ensure the freshness and comparable composition of both oils, fatty ester methyl ester (FAME) analysis was performed (Table 1). Long-chain fatty acids and very few degradation products were observed, which indicated freshness. High-oleic sunflower oil had a relative amount of 77% oleic ester, which is within the expected range for this specification.

Acid value determination via titration revealed 0.407 ± 0.081 mg/g for high-oleic sunflower oil and 0.410 ± 0.075 mg/g for palm oil, which confirmed that the oils were fresh when they were under the threshold of 0.6 mg KOH/g oil. Table 1 shows the fatty acid compositions of the oils used in the study and shows a comparison of the reference values for palm oil and high-oleic sunflower oil according to CODEX STAN 210-1999 [13]. All of the relative amounts correspond to the CODEX- values. Erucic acid, which can occur in high-oleic sunflower oil, was not detected in the utilized samples.

### 3.5. Advantages and Disadvantages of the Utilized Methods

**Figure A1:**
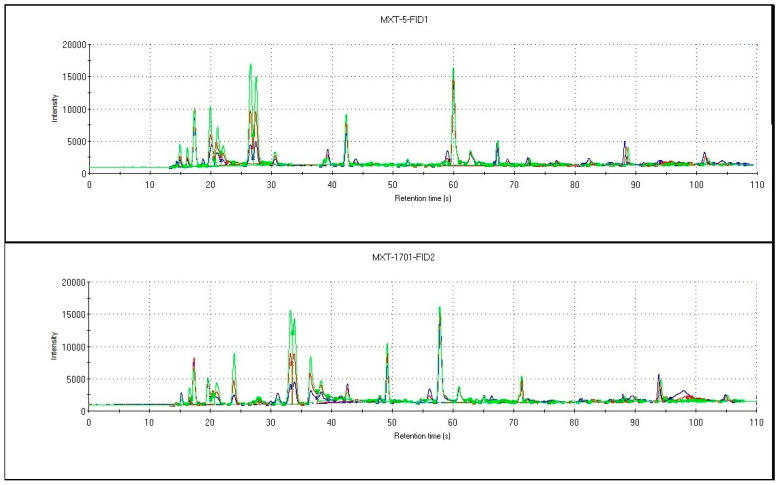
Overlay of the triplicate determinations of 2-week-old sugar/palm mixtures. The peaks were equal except for the height, which may be a result of some clumps that were formed during the incubation time, which led to a lower availability of volatile compounds.

All of the measurements using the electronic nose were performed in triplicate to ensure reproducibility. The overlay of the triplicates indicated a high similarity of the results. Although the variation in peak area and retention time for the electronic-nose measurements was considerably low among the triplicate measurements, substance identification based purely on the retention index alignment in the database proved to be irreproducible and prone to errors, as shown in the Appendix A (Table A1 and Figure A1). The sample in triplicate is shown as a chromatogram, and the substance identification for each graph is listed in Table A1, which shows that for a single peak, several substances were suggested, and the results of the replicates did not match.

**Figure A2:**
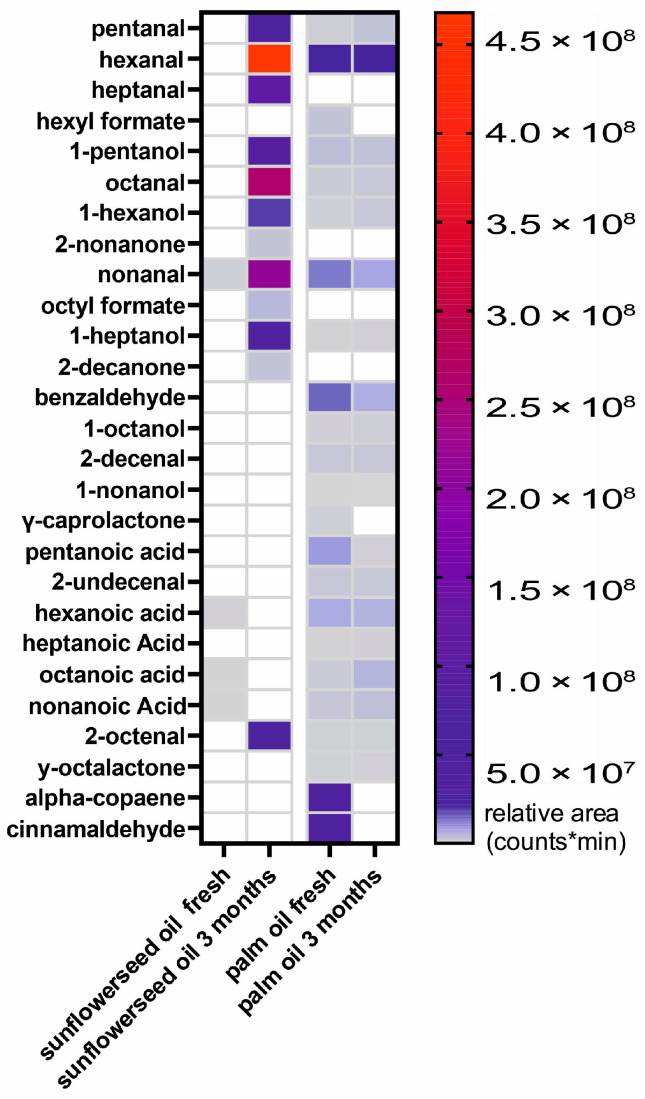
Heatmap of volatile compounds in the decorative sugar samples. Fresh and aged sugars with high-oleic sunflower-oil and palm-oil contents were measured with headspace GC-MS, and the relative amounts of the compounds are shown.

Aroma compounds for fresh and overstored sugar and oil mixtures were determined by using GC-MS (Appendix A Figure A2) to validate the findings of the electronic nose. Via the use of a mass spectrometer, as is the case for headspace GC-MS data, compounds can be tentatively identified based on their fragment ion mass spectra to improve comparability; this was not possible for the electronic nose, as identification relies solely on matching the Kovats retention index with the reference database values. A comparison of the identified substances of both measurements showed that the electronic-nose identification method is not suitable for complex food matrices such as decorative sugars.

However, the main application of electronic noses is the relative comparison of the aroma profiles of similar samples, rather than compound identification. The feature of database identification, wherein substances can be identified based on their Kovats retention index, is an additional tool, but this is not the dedicated function of the device.

Headspace GC-MS analyses were performed to compare the putative substances identified by the electronic nose with those determined using more reliable MS-based identification. Triplicates showed low standard deviations, which demonstrated high reproducibility. The measurements showed a greater increase in degradation products for high-oleic sunflower oil than for palm oil after 2.5 months of storage at 50 °C, as expected. The most abundant sunflower-oil degradation products were hexanal, heptanal, octanal, 1-pentanol, 1-hexanol, nonanal, 1-heptanol, and 2-octenal. Palm oil showed no degradation products after 2.5 months of storage; however, some compounds, such as alpha-copaene and cinnamaldehyde, were obviously lost. The samples were stored in headspace vials during the storage period. Therefore, we can conclude that the substances were not evaporated but rather degraded.

## 4. Discussion

We sought a high-throughput method to determine the suitability of different plant-based oils as a supplement for palm oil in decorative sugar mixtures due to the lower acceptance of these oils by consumers. Various sweet bakery products consumed in central Europe, such as Christmas cookies or fried carnival baked goods (Krapfen in Austria or chiacchere in Italy), are decorated with powdered sugar after baking or frying in hot oil. All of these methods necessitate different properties in the sugar coating. Therefore, a high-throughput method is needed to characterize the properties of various sugar and oil combinations for each product. Common saccharose is highly hygroscopic and therefore immediately melts, resulting in an uneven appearance. To prevent this melting process and to ensure a high degree of flowability, flow aids such as starch or small amounts of vegetable oil are used. Due to changes in eating behavior and the awareness of palm oil being present in many goods, the bakery industry needs to substitute palm oil for a more accepted alternative. We investigated the impact of various oils on powdered decorative sugars and compared technological alternatives to classical and laborious acid-value determinations via Soxhlet extraction to determine storage stability. There is only a small percentage amount of oil in powdered decorative sugars. Nevertheless, the impact of the oil on the stability, taste and physical properties should not be neglected.

The respective performance of a multispectral imaging device and an electronic nose for determining volatile compounds was compared to identify high-throughput methods. The advantage of multispectral imaging is the simplicity of sample preparation; the only requirement is that the surface should be as even as possible to avoid shadows from larger particles. Multispectral imaging is a nondestructive detection system suitable for a broad range of food quality and safety determinations [11,14,15,16]. As soon as the software is trained using appropriate control samples, a session can be programmed, and all other data are subsequently evaluated within a few seconds. The results are displayed in an Excel table without any further calculations. Inhomogeneous samples can be recognized using multispectral imaging, which is not the case for electronic noses. A shortcoming of visual inspection is that each sample must be placed under the sphere of the VideometerLab device separately, which requires more working time on the device than does the electronic nose. Another shortcoming is that one must be careful with transparent samples such as pure oils. A transparent oil sample will show the background, but this effect can be standardized; for example, a 99% reflective plate can be used, which will focus the measurement on absorption, whereas a black-background plate will focus the measurement on scattering. Thus, it is not a problem that the background can be specified to focus on the targeted phenomena, as long as it is standardized in the protocol.

Food color can also be a problem for spectral analysis, and some other additives can shift the absorption at specific wavelengths. However, minimizing the interference of different chromophores/absorbents is inherent in reflectance/absorbance spectroscopy and should be reduced in the analysis phase by optimizing the signal/noise ratio. Subsequently, the signal represents a variation caused by relevant changes, and noise is a variation caused by other additives.

The advantage of the electronic nose is that as soon as the software is trained with good and bad samples, it can be programmed to set a threshold of acceptable quality parameters. Untrained users can add their samples to the tray and load a routine, and the device provides a yes/no answer concerning the quality of the product. Data measurements require just a few minutes of analysis per sample, which is much faster than a standard GC-MS measurement. The disadvantages include the fact that the data evaluation is not easy to perform if the relevant substance is unknown. Especially in this case, the identification of the off-flavors was not possible, as the identification capabilities solely based on retention time are limited. Furthermore, a critical parameter for obtaining reproducible and comparable data is the use of the same sample weight. Being slightly less precise and using a sample that only slightly differs from the others can produce large variations in the results. The measurement technique is also destructive, to some degree. Furthermore, the sample is still present after measurement, but it must be heated to release as many volatile compounds as possible. Therefore, it is not possible to remeasure a sample and obtain the same results.

In previous studies, both devices were used for food- and feed-quality control. Multispectral imaging was used for seed-quality classification [16,17,18], observing the fermentation status of water kefir [19], performing quality assessment in butter cookies [11,20] or identifying adulteration in coffee [21] or meat [22]. Nondestructive measurements were also performed on lean pork slices to successfully identify bone fragments [14].

The studies of volatile compounds in food via electronic-nose measurements include successful determination of the freshness of bananas [23], the ripening stage of mangoes [24] and bacterial spoilage of chicken meats [25], although limitations have been reported, such as in red wine spoilage [26] or wine-must fermentation [27]. The electronic nose is a promising tool for food quality assessment. In this study, the electronic nose delivered the expected values and its suitability for rancidity detection was demonstrated.

## 5. Conclusions

Both devices have their advantages and shortcomings. The multispectral device VideometerLab is easy to use and delivers fast reproducible results, although each sample must be placed individually under a sphere for measurement. The electronic nose Heracles is easy to operate, although one must be very precise with sample preparation, as slight weight differences may result in large standard deviations. Both devices are used in the food industry and are being continually developed and adapted to specific issues. In summary, our study confirms that high-oleic acid sunflower seed oil is an appropriate alternative to palm oil, limited only by a slightly lower storage stability.

## Figures and Tables

**Table 1:** Fatty acid methyl ester analysis of fresh high-oleic sunflower and palm oils and reference values from CODEX STAN 210-1999.

	Relative Share (%)				CODEX STAN 210-1999		
	High-Oleic Sunflower Oil		Palm Oil			High-Oleic Sunflower Oil	Palm Oil
	AV	*Stdev*	AV	*Stdev*			
Methyl Odecanoate (C12:0)	ND		ND		C12:0	ND	ND–0.5
Methyl Myristate (C14:0)	0.07	0.00	1.38	0.03	C14:0	ND–0.1	0.5–2.0
Methyl Palmitate (C16:0)	4.24	0.04	46.70	0.74	C16:0	2.6–5.0	39.3–47.5
Methyl Palmitoleate (C16:1, n-7)	0.12	0.00	0.16	0.00	C16:1	ND–0.1	ND–0.6
Methyl Heptadecanoate (C17:0)	0.05	0.00	0.15	0.00	C17:0	ND–0.1	ND–0.2
cis-10-Heptadecanoic acid methyl ester (C17:1, n-7)	ND		ND		C17:1	ND–0.1	ND
Methyl Stearate (C18:0)	3.06	0.05	5.39	0.08	C18:0	2.9–6.2	3.5–6.0
cis-9-Oleic Acid Methyl Ester (C18:1, n-9)	76.82	0.53	36.84	0.49	C18:1	75–90.7	36.0–44.0
cis-11-Vaccenic Acid Methyl Ester (C18:1, n-7)	ND		0.82	0.03			
Methyl Linoleate (C18:2, n-6)	13.49	0.43	7.59	0.17	C18:2	2.1–17	9.0–12.0
Methyl y-Linolenate (C18:3, n-6)	ND		0.04	0.00	C18:3	ND–0.3	ND–0.5
Methyl Linoleate (C18:3, n-3)	0.35	0.01	0.20	0.01	C20:0	0.2–0.5	ND–1.0
Methyl Arachidate (Eicosanoate C20:0)	0.29	0.01	0.44	0.01	C20:1	0.1–0.5	ND–0.4
Methyl-cis-11-Eicosenoate (Gondoate, C20:1)	0.32	0.00	0.12	0.01			
Methyl Behenate (C22:0)	0.87	0.00	0.09	0.00	C22:0	0.5–1.6	ND–0.2
Methyl Eruate (C22:1, n-9)	ND		ND		C22:1	ND–0.3	ND
cis-13,16-Docosadienoic Acid Methyl Ester (C22:2, n-6)	ND		ND		C22:2	ND	ND
Methyl Lignocerate (C24:0)	0.32	0.01	0.09	0.00	C24:0	ND–0.5	ND
ND-non detectable, defined as ≤0.05%							

## Data Availability

The original contributions presented in the study are included in the article, further inquiries can be directed to the corresponding author.

## Appendix A

**Table A1:** Compounds determined from 2-week-old sugar/palm mixtures. The relevance index was >97, but the identification of the substance was not clear.

	Retention Time MXT-5-FID1	Retention Index MXT-5-FID1	Retention Time MXT-1701-FID2	Retention Index MXT-1701-FID2	Formula	Name	Sensory Descriptors
first sample	26.52	654	33.25	742	C_5_H_10_O	3-methylbutanal	Aldehydic; Almond; Apple; Cheese; Chocolate; Fatty; Fruity; Green; Herbaceous; Malty; Peach; Toasted
	26.52	654	36.59	773	C_4_H_10_O	n-butanol	Alcoholic; Amyl alcohol; Banana; Cheese; Fermented; Fruity; Fusel; Harsh; Medicinal; Oil; Rancid; Strong; Sweet
	42.34	803	49.21	894	C_6_H_12_O	Hexanal	Acorn; Aldehydic; Fatty; Fishy; Fresh; Fruity; Grassy; Green; Herbaceous; Leafy; Sharp; Strong; Sweaty; Tallowy; Vinous
	72.84	1209	76.05	1300	C_10_H_20_O	Decanal	Aldehydic; Burnt; Citrus; Fatty; Floral; Green; Herbaceous; Lemon; Orange; Orange peel; Soapy; Stew; Sweet; Tallowy; Waxy
	81.1	1369	88.53	1566	C_9_H_16_O_2_	gamma-nonalactone	Coconut; Creamy; Fruity; Oily; Peach; Strong; Sweet; Waxy; Woody
	82.28	1393	89.47	1588	C_11_H_14_O_2_	Methyl eugenol	Carnation; Cinnamon; Clove; Fresh; Mild; Spicy; Sweet; Warm
	95.86	1694	100.65	1843	C_13_H_10_O	Benzophenone	Balsamic; Geranium; Metallic; Powdery; Powdery (faint); Rose
second sample	22.96	612	27.76	689	C_4_H_8_O_2_	Ethyl Acetate	Acidic; Butter; Caramelized; Ethereal; Fragrant; Fruity; Green; Orange; Pineapple; Pungent; Solvent; Sweet
	26.52	654	33.2	742	C_5_H_10_O	3-methylbutanal	Aldehydic; Almond; Apple; Cheese; Chocolate; Fatty; Fruity; Green; Herbaceous; Malty; Peach; Toasted
	42.26	803	49.16	893	C_6_H_12_O	Hexanal	Acorn; Aldehydic; Fatty; Fishy; Fresh; Fruity; Grassy; Green; Herbaceous; Leafy; Sharp; Strong; Sweaty; Tallowy; Vinous
	42.26	803	49.16	893	C_6_H_12_O_2_	Butyl acetate	Banana; Bitter; Ethereal; Fruity; Green; Pear; Pineapple; Pleasant; Solvent; Strong; Sweaty; Sweet
	58.18	976	63.9	1083	C_7_H_6_O	Benzaldehyde	Almond; Bitter; Bitter almond; Burnt sugar; Cherry; Fruity; Malty; Oil; Pepper; Sharp; Strong; Sweet; Woody
	61.48	1022	65.12	1101	C_8_H_16_O	Octanal	Aldehydic; Citrus; Fatty; Floral; Fruity; Green; Lemon; Meat (boiled); Orange; Orange peel; Pungent; Rancid; Soapy; Stew; Strong; Waxy
	61.48	1022	65.12	1101	C_10_H_16_	alpha-Terpinene	Citrus; Ethereal; Fruity; Gasoline; Lemon; Medicinal; Woody
	61.48	1022	65.12	1101	C_8_H_14_O_2_	trans-hex-2-enyl acetate	Apple; Banana; Fresh; Green; Sweet; Waxy
	65.8	1087	69.2	1172	C_9_H_18_O_2_	Pentyl butanoate	Apricot; Banana; Cherry; Fruity; Fruity (sweet); Pineapple; Sweet; Tropical
	65.8	1087	69.2	1172	C_8_H_12_N_2_	Tetramethylpyrazine	Burnt; Chocolate; Cocoa; Coffee; Grassy; Hay; Musty; Nutty; Roast
	65.8	1087	69.2	1172	C_9_H_18_O	nonan-2-one	Baked; Cheese; Earthy; Fatty; Fresh; Fruity; Green; Ketonic; Milk (hot); Musty; Soapy; Sweet; Varnish
	65.8	1087	69.2	1172	C_9_H_18_O_2_	ethyl heptanoate	Fruity
	65.8	1087	69.2	1172	C_8_H_18_O	1-Octanol	Aldehydic; Bread (toasted); Burnt matches; Chemical; Fatty; Floral; Fresh; Green; Herbaceous; Metallic; Mushroom; Orange; Rose; Sulfurous; Waxy
	73.02	1213	76.24	1303	C_10_H_20_O	Decanal	Aldehydic; Burnt; Citrus; Fatty; Floral; Green; Herbaceous; Lemon; Orange; Orange peel; Soapy; Stew; Sweet; Tallowy; Waxy
	74.56	1242	76.84	1316	C_10_H_20_O	Decanal	Aldehydic; Burnt; Citrus; Fatty; Floral; Green; Herbaceous; Lemon; Orange; Orange peel; Soapy; Stew; Sweet; Tallowy; Waxy
	76.16	1272	88.1	1557	C_8_H_7_N	indole	Animal; Burnt; Earthy; Fecal; Floral; Jasmine; Mothball; Musty; Sweet
	81.38	1375	86.1	1511	C_10_H_12_O_2_	Eugenol	Balsamic; Camphor; Clove; Floral; Herbaceous; Honey; Spicy; Sweet; Warm; Woody
	92.56	1619	101.5	1863	C_11_H_20_O_2_	delta-Undecalactone	Coconut; Creamy; Fatty; Fruity; Peach; Waxy
third sample	42.29	803	49.16	893	C_6_H_12_O	Hexanal	Acorn; Aldehydic; Fatty; Fishy; Fresh; Fruity; Grassy; Green; Herbaceous; Leafy; Sharp; Strong; Sweaty; Tallowy; Vinous
	58.13	976	63.86	1083	C_7_H_6_O	Benzaldehyde	Almond; Bitter; Bitter almond; Burnt sugar; Cherry; Fruity; Malty; Oil; Pepper; Sharp; Strong; Sweet; Woody
	61.49	1022	65.1	1101	C_8_H_16_O	Octanal	Aldehydic; Citrus; Fatty; Floral; Fruity; Green; Lemon; Meat (boiled); Orange; Orange peel; Pungent; Rancid; Soapy; Stew; Strong; Waxy
	61.49	1022	65.1	1101	C_10_H_16_	alpha-Terpinene	Citrus; Ethereal; Fruity; Gasoline; Lemon; Medicinal; Woody
	61.49	1022	65.1	1101	C_8_H_14_O_2_	trans-hex-2-enyl acetate	Apple; Banana; Fresh; Green; Sweet; Waxy
	73.17	1216	76.2	1303	C_10_H_20_O	Decanal	Aldehydic; Burnt; Citrus; Fatty; Floral; Green; Herbaceous; Lemon; Orange; Orange peel; Soapy; Stew; Sweet; Tallowy; Waxy
	73.85	1228	78.38	1347	C_10_H_18_O	Nerol	Citrus; Floral; Magnolia; Marine; Rose; Sweet
	73.85	1228	78.38	1347	C_9_H_14_O	(E, E)-2,4-Nonadienal	Cereal; Cucumber; Deep-fried; Fatty; Fried; Green; Melon; Oily; Potato; Soapy; Tropical; Violet; Watermelon; Waxy; Wool (wet)
	92.89	1627	100.56	1841	C_11_H_20_O_2_	delta-Undecalactone	Coconut; Creamy; Fatty; Fruity; Peach; Waxy
	95.71	1691	100.56	1841	C_13_H_10_O	Benzophenone	Balsamic; Geranium; Metallic; Powdery; Powdery (faint); Rose
